# A successfully thrombolysed acute inferior myocardial infarction due to type A aortic dissection with lethal consequences: the importance of early cardiac echocardiography

**DOI:** 10.1186/1749-8090-6-101

**Published:** 2011-08-24

**Authors:** Grigorios Tsigkas, Georgios Kasimis, Konstantinos Theodoropoulos, Konstantinos Chouchoulis, Nikolaos G Baikoussis, Efstratios Apostolakis, Eleni Bousoula, Athanasios Moulias, Dimitrios Alexopoulos

**Affiliations:** 1 Department of Cardiology, Patras University School of Medicine, Patras, Greece; 2 Department of Cardiothoracic Surgery, Patras University School of Medicine, Patras, Greece

## Abstract

Thrombolysis, a standard therapy for ST elevation myocardial infarction (STEMI) in non-PCI-capable hospitals, may be catastrophic for patients with aortic dissection leading to further expansion, rupture and uncontrolled bleeding. Stanford type A aortic dissection, rarely may mimic myocardial infarction. We report a case of a patient with an inferior STEMI thrombolysed with tenecteplase and followed by clinical and electrocardiographic evidence of successful reperfusion, which was found later to be a lethal acute aortic dissection. Prognostic implications of early diagnosis applying transthoracic echocardiography (TTE) are described.

## Background

Acute myocardial infarction (AMI) usually results from an occlusive coronary thrombus at the site of a ruptured atherosclerotic plaque [1]. Reperfusion therapies such as primary percutaneous coronary intervention (PPCI) and thrombolysis are mandatory steps for reducing mortality and limiting the infarct size in patients with ST segment elevation myocardial infarction (STEMI). The greatest benefit occurs, if reperfusion therapy is initiated within the first hours from the onset of symptoms and there is no preference for either strategy, if these symptoms are present for less than 3 hours [2]. Clinically speaking, many conditions, such as acute aortic dissection, pericarditis, pulmonary embolism and myocarditis may mimic acute myocardial infarction. Thrombolysis in most of these situations is absolutely contraindicated due to its potentially lethal complications. Clinicians should always bear in mind the possibility that a type A aortic dissection (AAD) may mimic an AMI, which requires an urgent surgical repair without any delay.

## Case presentation

**Figure 1:**
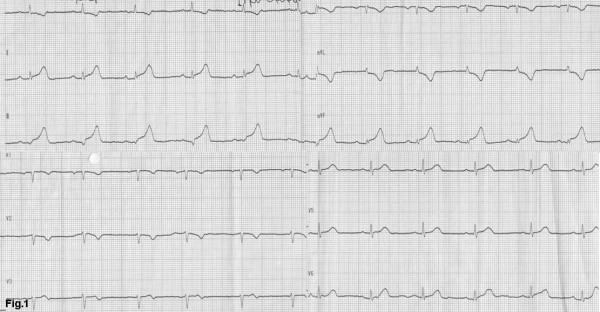
**The initial electrocardiogram (ECG): ECG shows sinus rhythm with ST elevation in leads II, III, aVF and reciprocal changes in I, aVL**.

**Figure 2:**
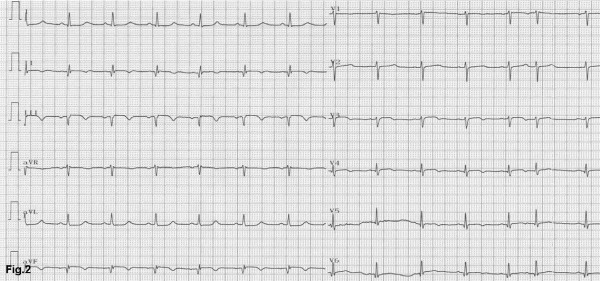
**Post thrombolysis ECG: ECG at our ED, with sinus rhythm, Q and negative T waves at the inferior leads, premature atrial contractions and non specific secondary changes of ST at the lateral wall**.

**Figure 3:**
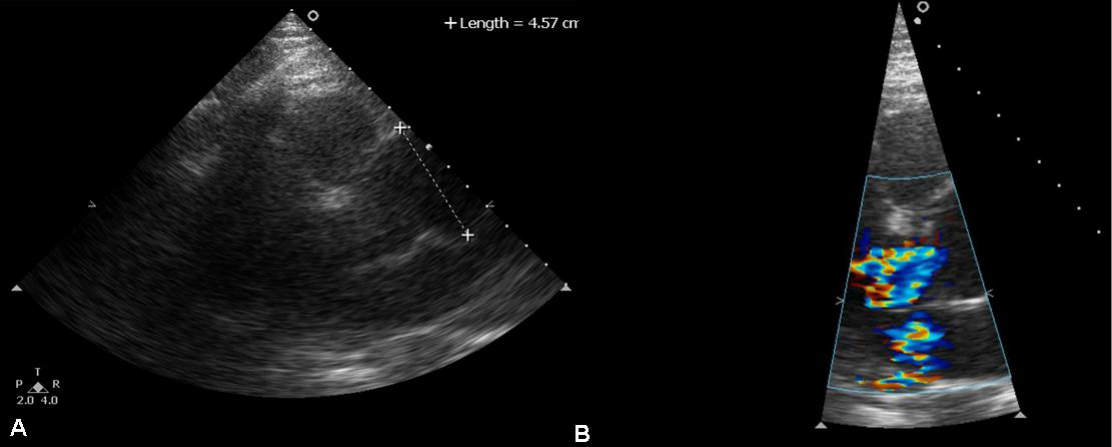
**Transthoracic echocardiography (TTE): Panel A depicts long axis parasternal view with a dilated aortic root of 4.57 cm, without pericardial effusion**. Panel B the use of Color Flow Doppler unveiled a moderate aortic and mild mitral regurgitation.

**Figure 4:**
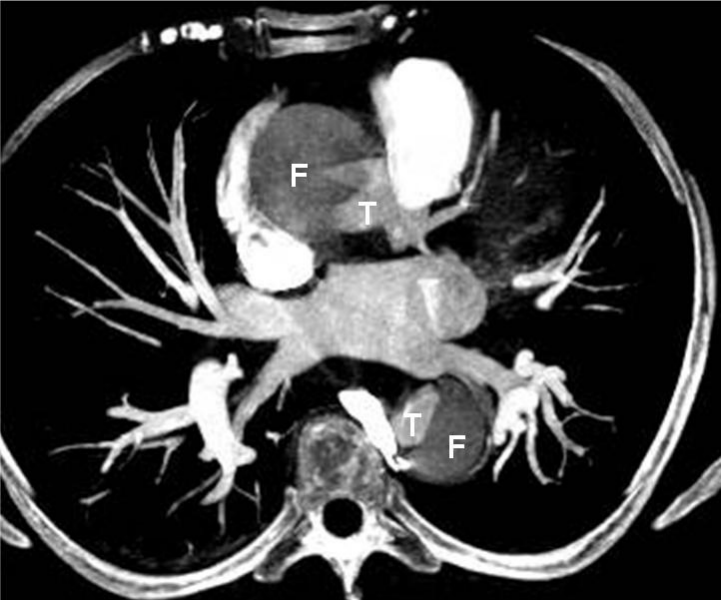
**Multidetector Computed Tomography Angiography (MDCTA): Axial plane demonstrates an intimal flap that separates the false (F) from the true lumen (T) in the ascending and descending aorta, diagnostic of a Stanford type A dissection**.

**Figure 5:**
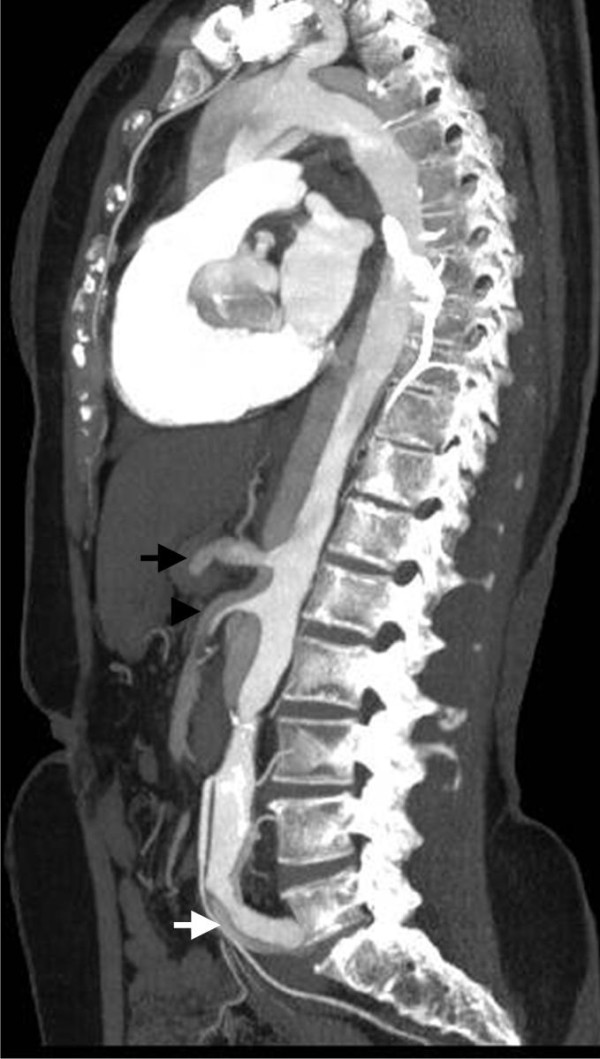
**Multidetector Computed Tomography Angiography (MDCTA): Median plane depicts the extended dissection from the ascending aorta, passing through the origins of celiac trunk (black arrow) and superior mesenteric artery (arrow head) down to the iliac arteries (white arrow)**.

**Figure 6:**
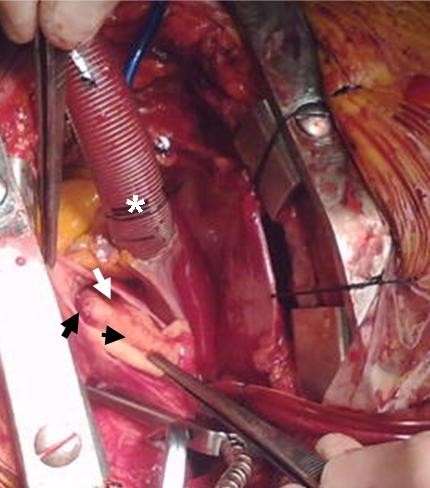
**During operation: The origin of the right coronary artery from the false lumen (black arrow), the false lumen (white arrow), the intimal flap (arrow head) and the venous cannula inserted in the right atrium are seen (asterisk)**.

**Figure 7:**
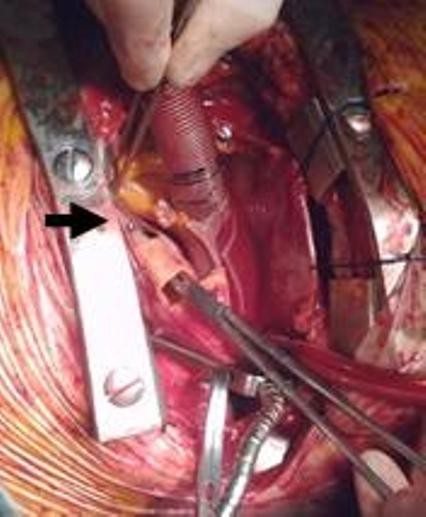
**During operation: A real transection of the right coronary artery (black arrow) is shown**. A dissector was passed through the coronary ostium till the side of its rupture.

A 57-year-old woman, with a history of hypertension, was admitted to the emergency department of a rural non-PCI-capable Hospital due to an atypical, non-compressing, non-excruciating chest pain of recent origin (30 minutes) with radiation to the back. The patient was hemodynamically stable, with no peripheral pulse deficit. Auscultation of the heart revealed a 2/6 systolic murmur at the right base and apex and an early diastolic murmur at the right base without pericardial friction. The electrocardiogram (ECG) (Figure 1) was compatible with the diagnosis of a STEMI of the inferior wall. The doctor in charge decided to administer thrombolytic treatment with tenecteplase (TNK) (Metalyse^®^) without further delay. The patient's symptoms were partially relieved, while the pre-existing ST elevation did not seem to be completely normalized in the following 60 minutes. For this reason, she was referred to our hospital for rescue angioplasty [3]. When the patient arrived had a remission both of the initial thoracic pain and of ST-elevation in the ECG (Figure 2), remaining hemodynamically stable without obvious perceivable peripheral artery pulse deficit, but was clearly uncomfortable and she was complaining for a diffuse abdominal pain without any sign of peritoneal irritation. In addition, she was anuric during the whole time of her transport, namely about 3 hours. Blood sample analysis showed: Hct 37%, WBC 14,600/μL, PMN 86%, Ur 70 mg/dl, Cr 1.3 mg/dl, SGOT 63 U/L, CPK 483 U/L, TNI 2.1 ng/ml. A bedside chest x-ray was not diagnostic, while a quick bedside transthoracic echocardiography (TTE) revealed severe hypokinesia of the posterior and the inferior wall and a dilated aorta with a high suspicion of an intimal dissection flap (Figure 3a). Color flow Doppler showed moderate aortic and mild mitral regurgitation (Figure 3b). AAD complicated with an inferior AMI was highly suspected. The following Multidetector Computed Tomography Angiography (MDCTA) confirmed the extended dissection from the ascending aorta to the iliac arteries (Figure 4 and 5). Cardiac surgeons were immediately informed and an emergent replacement of the aortic root and ascending aorta was decided. The patient was transported in the operating room in a critical state. The right axillary artery was cannulated before sternotomy. Then, a median sternotomy was performed and the pericardium was opened. A dilated ascending aorta, clots in the pericardium, dilatation of the right cardiac chambers due to infarction and a sub-epicardial hematoma along the right coronary artery in the atrio-ventricular groove were found. The right atrium was cannulated, the distal aorta was cross clamped and cold blood-crystalloid (4/1) cardioplegia was administrated initially retrograde via the coronary sinus and then anterograde via the left main coronary artery. The procedure took place under systematic hypothermia (25°C).. After the opening of the aorta the diagnosis of a type A aortic dissection was confirmed with the implication of the right coronary artery for its first 5 mm (Figure 6). A rare finding was the real transection-disruption of the right coronary artery; the cause of the acute myocardial infarction (Figure 7). A Bentall procedure was performed with the implantation of a valved-graft. A coronary artery bypass grafting with a saphenous vein graft anastomosed in the right coronary artery at the level of the crux was also done. The patient was rewarmed and weaned successfully from the cardiopulmonary bypass. Unfortunately, the patient died 48 hours after the operation because of multiple organ failure.

## Discussion

Thrombolysis is currently recommended for patients with STEMI presenting in non-PCI-capable centers, especially when PCI in less than 2 h transfer is not possible [2]. Patients with similar symptoms, but without myocardial infarction, may be falsely treated with thrombolysis. Acute ascending aortic dissection associated with AMI is rare, with a reported incidence of 1 to 2% [4]. In previous reports [5,6], most patients with aortic dissection, who were treated with thrombolytic agents died due to hemorrhagic complications. The non-invasive identification of successful fibrinolysis remains a challenging issue, using pain cessation and more than 50 per cent ST-segment resolution in the lead(s) with the highest ST-segment elevations 60 to 90 minutes after initiation of fibrinolytic therapy as a useful surrogate [3]. Although, the primary event, which caused AMI was aortic dissection, two hypotheses may explain the coronary occlusion. Since the dissection can partially occlude the ostium of a coronary artery, it can modify the blood flow and pressure inside the vessel, leading to coronary thrombosis and consequent myocardial infarction. Tenekteplase, by dissolving the thrombi through the false lumen of the coronary artery and aortic root, allowed reperfusion, but later led to further bleeding and occlusion [7]. Another possibility is that a coronary spasm was the cause of the STEMI, due to nearby hematoma or pressure of the false lumen which resolved with the use of the adjuvant therapy, such as nitrates. The followed inappropriate thrombolysis probably affected adversely the outcome by being the causative factor for the further expansion of the tear and by causing difficulties with the hemostasis at the surgery [8,9]. Aortic dissection is caused by an intimal tear following elastic degeneration, smooth muscle cells loss or elevated pressure in vasa vasorum, which leads to rupture and allows the creation of a false lumen between the media and adventitia [10]. The presence of severe chest pain of very sudden onset, usually described as tearing and followed from a feeling of impending death, a discrepancy in the pulse or blood pressure in the two upper extremities or between upper and lower extremities and a widening of mediastinal on chest X-ray are reported to have a probability of 96% for the diagnosis of AAD. On the other hand, if those signs are not present, the probability of AAD is only 7% [11]. Sometimes is difficult to distinguish AAD from angina pectoris. Other common presentations for type A dissections include syncope (13% of type A AADs) and abdominal pain (22% of type A AADs and 43% of type B AADs). The above symptoms have important prognostic implications, signaling increased risks for shock, ischemia or infarction complications of mesenteric and limb arteries and in-hospital mortality, mainly because of delay in diagnosis [12]. Most of the patients with AAD, about 75%, have a history of hypertension [13]. Aortic dissection is less common than myocardial infarction and its association with ST segment elevation is unusual [14,15]. This combination can be recognized early with the help of diagnostic imaging, minimizing the risk of thrombolysis in selected patients. Bedside chest X-ray is not sufficient to rule out aortic dissection, but a great percentage of patients with AAD have an abnormal one, often showing a distended aorta or generalized widening of the mediastinum [16]. Transthoracic followed by transesophageal echocardiography (TEE), MDCTA and Magnetic Resonance Imaging (MRI) are very important and essential imaging tools with high sensitivity and specificity for early life-saving diagnosis of aortic dissection [4]. The TTE is of great importance because it is an easy, non-invasive, widely available and minimally time consuming technique, which can play a major role in differential diagnosis in emergency department. It can provide much information about possible aortic dilation and insufficiency of the aortic valve, pericardial and/or pleural effusion and finally about a dissection flap, which is the hallmark for the diagnosis of the aortic dissection. Despite that a remarkable improvement in development of new biomarkers has been made, there is no widely accepted strategy in this field. The biochemical diagnosis of aortic dissection has become possible by identifying raised concentrations of smooth muscle myosin heavy chain [17]. More recently, widely available biomarkers, such as D-dimer are thought to play an assistive role [18]. Our knowledge for the identification and the management of acute aortic pathology has made a tremendously improvement, mainly due to International Registry of Acute Aortic Dissection (IRAD). It is now known that if type A AAD remains untreated, one third of patients die within the first 24 hours, and the half of them die within 48 hours. According to latest data, surgery is the best option with a mortality rate of 5 to 21% for type A AAD and medication only is the best choice for an uncomplicated type B AAD with a mortality rate of approximately 20% [19].

## Conclusions

Thrombolytic treatment for STEMI, whenever PCI is not available, should not be postponed, except in cases of suspected aortic dissection. Our case shows that even clinical and electrocardiographic signs of successful reperfusion can occur when aortic dissection is the primary cause of the myocardial infarction. The presence of eccentric aortic regurgitation, the dilated ascending aorta and the possible visualization of double lumen by TTE could provide strong hints of the coexistence of AMI and type A aortic dissection. In conclusion, if aortic dissection is suspected, arrangement of the appropriate imaging studies should be done without further delay. Hence, bedside TTE can help as it is an easy, safe and rapid procedure to diagnose proximal aortic dissection without crucial delay. Cardiologists should bear in mind this usually lethal complication of acute aortic dissection and perform TTE prior to catheterization and even more before fibrinolysis.

## Consent

Written informed consent was obtained from the next of kin of the deceased patient for publication of this case report and the accompanying images. A copy of the written consent is available for review by the Editor-in-Chief of this journal.

## Competing interests

The authors declare that they have no competing interests.

## Authors' contributions

GT has made substantial contributions to conception and design, has been involved in drafting the manuscript and revising it critically for important intellectual content, GK has been involved in drafting the manuscript, KT carried out the echocardiogram studies and has made substantial contributions of data analysis, KC has made substantial contributions to conception and design of the manuscript, NB participated in the operation, EA performed the operation, EB has been involved in interpretation of echocardiogram and has made substantial contributions of data analysis, AM has made substantial contributions to design the manuscript and DA has made substantial contribution to design and has given the final approval of the version to be published. All authors read and approved the final manuscript.
